# Deficiency of SDHC promotes metastasis by reprogramming fatty acid metabolism in colorectal cancer

**DOI:** 10.1186/s12967-024-05361-x

**Published:** 2024-06-06

**Authors:** Zhuoyu Ding, Yiyi Wei, Jingping Dai, Chaomin Pan, Li Yang, Qingyuan Li, Yue Zhang, Qun Yan, Changjie Wu, Aimin Li, Zhixian Lan, Side Liu, Xinke Wang

**Affiliations:** 1 Guangdong Provincial Key Laboratory of Gastroenterology, Department of Gastroenterology, Nanfang Hospital, Southern Medical University, Guangzhou, China; 2 Pazhou Lab, Guangzhou, Guangdong China; 3 State Key Laboratory of Organ Failure Research, Guangdong Provincial Key Laboratory of Viral Hepatitis Research, Department of Infectious Diseases, Nanfang Hospital, Southern Medical University, Guangzhou, China

**Keywords:** SDHC, Metastasis, Colorectal cancer, Fatty acid metabolism

## Abstract

**Background:**

Several studies have demonstrated a strong correlation between impaired Succinate dehydrogenase (SDH) function and the advancement of tumors. As a subunit of SDH, succinate dehydrogenase complex subunit C (SDHC) has been revealed to play tumor suppressive roles in several cancers, while its specific role in colorectal cancer (CRC) still needs further investigation.

**Methods:**

Online database were utilized to investigate the expression of SDHC in colorectal cancer and to assess its correlation with patient prognosis. Cell metastasis was assessed using transwell and wound healing assays, while tumor metastasis was studied in a nude mice model in vivo. Drug screening and RNA sequencing were carried out to reveal the tumor suppressor mechanism of SDHC. Triglycerides, neutral lipids and fatty acid oxidation were measured using the Triglyceride Assay Kit, BODIPY 493/503 and Colorimetric Fatty Acid Oxidation Rate Assay Kit, respectively. The expression levels of enzymes involved in fatty acid metabolism and the PI3K/AKT signaling pathway were determined by quantitative real-time PCR and western blot.

**Results:**

Downregulation of SDHC was found to be closely associated with a poor prognosis in CRC. SDHC knockdown promoted CRC metastasis both in vitro and in vivo. Through drug screening and Gene set enrichment analysis, it was discovered that SDHC downregulation was positively associated with the fatty acid metabolism pathways significantly. The effects of SDHC silencing on metastasis were reversed when fatty acid synthesis was blocked. Subsequent experiments revealed that SDHC silencing activated the PI3K/AKT signaling axis, leading to lipid accumulation by upregulating the expression of aldehyde dehydrogenase 3 family member A2 (ALDH3A2) and reduction of fatty acid oxidation rate by suppressing the expression of acyl-coenzyme A oxidase 1 (ACOX1) and carnitine palmitoyltransferase 1A (CPT1A).

**Conclusions:**

SDHC deficiency could potentially enhance CRC metastasis by modulating the PI3K/AKT pathways and reprogramming lipid metabolism.

**Supplementary Information:**

The online version contains supplementary material available at 10.1186/s12967-024-05361-x.

## Introduction

Colorectal cancer (CRC) is one of the most common malignant tumors, with the third highest incidence rate and the second highest mortality rate [1]. The 5-year survival rate of early-stage cancer can be as high as 90%, while the survival rate for patients with advanced colorectal cancer is less than 10% due to tumor metastasis, chemotherapy resistance and other complications [2]. Therefore, it is imperative to study the underlying molecular mechanisms associated with the metastasis of CRC in order to identify novel targets and develop new therapies.

The tricarboxylic acid (TCA) cycle, a common pathway for the final oxidation of mitochondria, serves as the hub of the metabolic link between carbohydrates, lipids, and amino acids. Succinate dehydrogenase (SDH) is an enzyme that catalyzes the oxidation of succinic acid to fumarate in the TCA cycle. It is also known as respiratory complex II in the mitochondrial electron transport chain and consists of four subunits (SDHA, SDHB, SDHC, SDHD). SDHA and SDHB act as catalytic subunits, and SDHC and SDHD provide the binding site for ubiquinones [3]. Studies have shown that impaired SDH function caused by mutations in genes encoding SDH complexes is closely related to tumor genesis and progression [4]. For example, SDHC mutation is thought to lead to tumorigenesis through increased oxidative stress, which damages DNA structure in the nucleus [5]. In liver cancer, SDHC-associated defects promote multiplication and metastasis of hepatocellular carcinoma via the ROS/NFκB signaling pathway [6]. In breast cancer, downregulation of SDHC promote tumor development by promoting the occurrence of epithelial mesenchymal transformation and structural reconstruction of mitochondrial organelles [7]. In colorectal cancer, the lack of SDHB promotes TGFβ-mediated colorectal cancer invasion and metastasis through the transcriptional inhibitory complex SNAIL1-SMAD3/4 [8]. Additionally, miR-142-5p can promote the development of colorectal cancer by targeting SDHB [9]. However, there have been no studies reporting the role of SDHC in colorectal cancer, and its specific mechanism of action needs to be elucidated.

Tumor initiation and progression require the metabolic reprogramming of cancer cells. Alterations in energy metabolism are a hallmark of cancer during metastasis and therapeutic resistance [10, 11]. In cancer, lipid metabolism is often modified, and studies have demonstrated that inhibiting lipid peroxidation can enhance CRC liver colonization in mouse models [12]. ACOX1, a key enzyme in fatty acid β-oxidation, acts as a tumor suppressor, and the downregulation of ACOX1 promotes CRC progression [13]. Carnitine palmitoyl transferase 1 (CPT1) controls the rate-limiting step of fatty acid oxidation (FAO). The CPT1 family comprises three subtypes, CPT1A (liver type), CPT1B (muscle type) and CPT1C (brain type) [14]. Weakened CPT1A expression is critical for lipid accumulation to promote clear cell renal carcinoma development [15]. Therapeutics targeting lipid metabolism offer exciting opportunities for cancer therapy [16, 17]. However, full comprehension of the mechanisms behind lipid accumulation in CRC is still lacking.

In this study, we used GEO datasets, TCGA database and mitochondrial genes to identify the key molecule of tumor metastasis, SDHC. We investigated the expression of SDHC in CRC and analyzed its correlation with metastasis both in vitro and in vivo. To explore the potential mechanisms, we conducted drug screening and RNA sequencing, and found that SDHC may affect lipid metabolism through the PI3K/AKT signaling axis, thus regulating tumor metastasis.

## Materials and methods

### Bioinformatic analyses

Gene Expression Profiling Interactive Analysis (GEPIA), an online platform used for customizing and visualizing data using TCGA and GTEx data, was used to perform overall survival (OS) or disease free survival (DFS, also called relapse-free survival and RFS) analysis based on gene expression [18].

The University of Alabama at Birmingham Cancer data analysis Portal (UALCAN) is an effective website for online analysis and mining of cancer data based on the relevant cancer data in TCGA database [19]. We analyzed the expression of genes in CRC by UALCAN.

We used RNAseq technology to detect the mRNA expression profile of sh-SDHC HCT116 cells and scrambled control HCT116 cells. The Deseq2 R Package was used to identify DEGs between scrambled control groups and sh-SDHC groups using the following criteria: (i) |log2FC (sh-SDHC/scramble) |> 0; (ii) P < 0.05.

Gene Ontology (GO) is a community-based bioinformatics resource that provides information about gene product function using ontologies to represent biological knowledge which covers three aspects of biology: biological processes, cellular components, and molecular functions [20].

Kyoto Encyclopedia of Genes and Genomes (KEGG) is a knowledge base that stores high-level functions and utilities of biological systems [21]. We performed GO and KEGG on DEGs. P-value < 0.05 was considered significant.

Gene Set Enrichment Analysis (GSEA) is a threshold-free method that analyzes all genes based on their differential expression rank or other score without any prior gene filtering [22]. GSEA was used to carry out KEGG pathway enrichment analysis using all genes of the RNAseq data.

### Clinical tissue specimens

We also used clinical CRC specimens and paired peri-tumor tissues to detect the expression of SDHC. Clinical CRC specimens and paired normal tissues were collected from 62 patients who underwent surgical treatment for CRC at Nanfang Hospital of Southern Medical University after obtaining informed consent. A diagnosis of CRC was histopathologically confirmed for each patient sample. Cancer tissues and matched normal tissues were stored at – 80 ℃ until use. The protocols used in this study were approved by Nanfang hospital’s Protection of Human Subjects Committee.

### Cell culture, plasmid construction, lentiviral construction and cell transfections

The human normal colon epithelial cell line, human colorectal cancer cell lines, SDHC knockdown and control cell lines (sh-SDHC and sh-NC), SDHC overexpressing and empty vector cell lines (SDHC and EV) were described previously in detail [23].

### qRT-PCR

RNA extraction, reverse transcription as well as quantitative reverse transcription polymerase chain reaction (qRT-PCR) were performed as described previously [23]. The sequences of the primers used were as follows:

*SDHC* mRNA (sense): 5ʹ- CTGTTGCTGAGACACGTTGGT-3ʹ,

*SDHC* mRNA (antisense): 5ʹ- ACAGAGGACGGTTTGAACCTA-3ʹ,

*ACOX1* mRNA (sense): 5ʹ- ACTCGCAGCCAGCGTTATG-3ʹ,

*ACOX1* mRNA(antisense): 5ʹ-AGGGTCAGCGATGCCAAAC-3ʹ,

*CPT1A* (sense): 5ʹ-TCCAGTTGGCTTATCGTGGTG-3ʹ,

*CPT1A* (antisense): 5ʹ-TCCAGAGTCCGATTGATTTTTGC-3ʹ,

*ALDH3A2* (sense): 5ʹ- AAACCAGTTAAGAAGAACGTGCT-3ʹ,

*ALDH3A2* (antisense): 5ʹ- CGAAGGGGTAATTCCAAGCTC-3ʹ.

*β-actin* (sense): 5ʹ- CTCGCCTTTGCCGATCC -3ʹ,

*β-actin* (antisense): 5ʹ- GGGGTACTTCAGGGTGAGGA -3ʹ.

### Western blot analysis and immunohistochemistry analysis

Western blotting (WB) was performed as described previously [23]. The primary antibodies used were as follows: anti-β-actin (66009-1-Ig, Proteintech, 1:10000), anti-SDHC (ab155999, Abcam,1:10000), anti-ACOX1 (10957-1-AP, Proteintech, 1:1000), anti-CPT1A (15184-1-AP, Proteintech, 1:1000), anti-ALDH3A2 (15090-1-AP, Proteintech, 1:1000), anti-P-AKT (66444-1-Ig, Proteintech, 1:1000), anti-P-PI3K (4228, Cell Signaling, 1:1000), anti-PI3K (T40115, Abmart, 1:1000), and anti-AKT (T55561, Abmart, 1:1000). Image J software was employed to analyze relative protein expression.

For immunohistochemistry (IHC) analysis, CRC specimens were fixed with 10% formaldehyde and then embedded in paraffin. After preparing 4-μm-thick continuous paraffin sections, deparaffinization and antigen retrieval were performed following the manufacturer’s instructions. These sections were incubated with anti-SDHC (ab155999, 1:250, Abcam) antibodies. After incubated with secondary antibodies (PV-6001, ZSGB-BIO), the sections were visualized with a DAB chromogenic agent (ZLI-9017, ZSGB-BIO) and observed under a microscope.

### Transwell assay and Wound healing assay

Transwell assay and wound healing assay were performed as described previously [23].

### Application of inhibitors

For the application of the fatty acid synthetase inhibitor orlistat (T0686, TargetMol), a concentration of 20 μmol/L orlistat was used to treat CRC cells for 48 h when necessary. Additionally, the AKT inhibitor MK-2206 (T1952, TargetMol) was employed at a concentration of 1 μmol/L to treat CRC cells for 48 h when necessary.

### Drug screening and cell viability measurement.

Anti-cancer Metabolism Compound Library containing 237 anti-cancer metabolism compounds was purchased from TargetMol(L2130). Each compound was arranged in 384-well plates in an 8-dose format, ranging from 22.9 nM to 50 μM of final concentrations. HCT116-sh-NC or HCT116-sh-SDHC cells were plated at 2000 cells per well in these plates with a diluted compound library and incubated for 72 h. Cell survival was assessed using the Resazurin sodium salt assay (R8150, Solarbio) following the manufacturer’s instructions. A 1/10 volume of resazurin solution was added to the cell and incubated at 37 ℃ for 2 h in the dark. After incubation, the fluorescence intensity at Ex/Em of 530/590 nm was measured to analyze cell survival. Using GraphPad Prism 7, the IC50 values for each compound were calculated. The Selectivity Index (SI) was determined using the formula: SI = IC_50_^sh−SDHC^/IC_50_^sh−NC^. Compounds with an SI greater than 2 were chosen as potential candidates.

### Quantification of triacylglycerol and neutral lipids

For the quantitative estimation of triglycerides in cells, we used a Triglyceride Assay Kit (BC0625, Solarbio) following the manufacturer’s protocols. We applied the lipophilic fluorescence dye BODIPY 493/503(GC42959, Glpbio) to stain the neutral lipid droplets. Flow cytometry was conducted to quantify the neutral lipid content, while confocal microscopy was used for visualizing the staining. The nucleus was counterstained with DAPI (P0131, Beyotime). And we quantified the fluorescence intensity of lipid droplets and cell numbers using Image‐J software.

### FAO quantification

The mitochondria of cells were isolated using the Cell Mitochondria Isolation Kit (C3601, Beyotime). After measuring the protein concentration of the mitochondria, they were subjected to the FAO rate assay using the Colorimetric Fatty Acid Oxidation Rate Assay Kit (HL50679, Haling), following the manufacturer’s protocol.

### In vivo experiments

Male athymic 4-week-old BALB/c nude mice were purchased from the Central Laboratory of Animal Science, Nanfang Medical University and maintained in a specific pathogen-free facility. For the liver metastasis model, mice were divided into two groups (n = 4 for each group) and were anaesthetized with pentobarbital sodium by intraperitoneal injection. A 1-cm incision was formed on the left side and the spleen was separated, a total of 5 × 10^6^ HCT116-sh-SDHC or HCT116-sh-NC cells were suspended in 0.05 ml PBS and then injected into the spleen with an insulin needle. The spleen was returned to the abdominal cavity, and the wound was sutured. After 4 weeks, the mice were sacrificed, and their livers were removed and carefully dissected to evaluate the metastatic lesions.

In the in vivo experiments involving orlistat, mice were divided into three groups, with three mice in each group. A liver metastasis model was constructed. After 3 days, Orlistat was administered in a 100 μl vehicle, which consisted of 10% ethanol, 50% PEG 300, 5% tween80, and 35% normal saline. The animals received a dosage of 240 mg/kg/day of orlistat or a control solvent. After 4 weeks, the mice were sacrificed, and their livers were carefully dissected to evaluate the presence of metastatic lesions.

For lung metastasis model, mice were divided into two groups (n = 3 for each group) and 5 × 10^6^ HCT116-sh-SDHC or HCT116-sh-NC cells in 0.15 ml PBS were injected into the tail vein of male BALB/c nude mice. After 4 weeks, the mice were sacrificed, and their lungs were removed and carefully dissected to evaluate the metastatic lesions. Animals was approved by the Nanfang hospital animal ethic committee.

### Statistical analysis

SPSS 21.0 statistical analysis software was employed for statistical analysis of the experimental data. Quantitative data in this study were presented as mean ± standard deviation from at least three replicates were analyzed by the two-tailed unpaired Student's t-test to compare the difference between groups. Significant differences were displayed as follows: *P < 0.05, **P < 0.01, and ***P < 0.001.

## Results

### Expression and prognostic value of SDHC in CRC

**Fig. 1:**
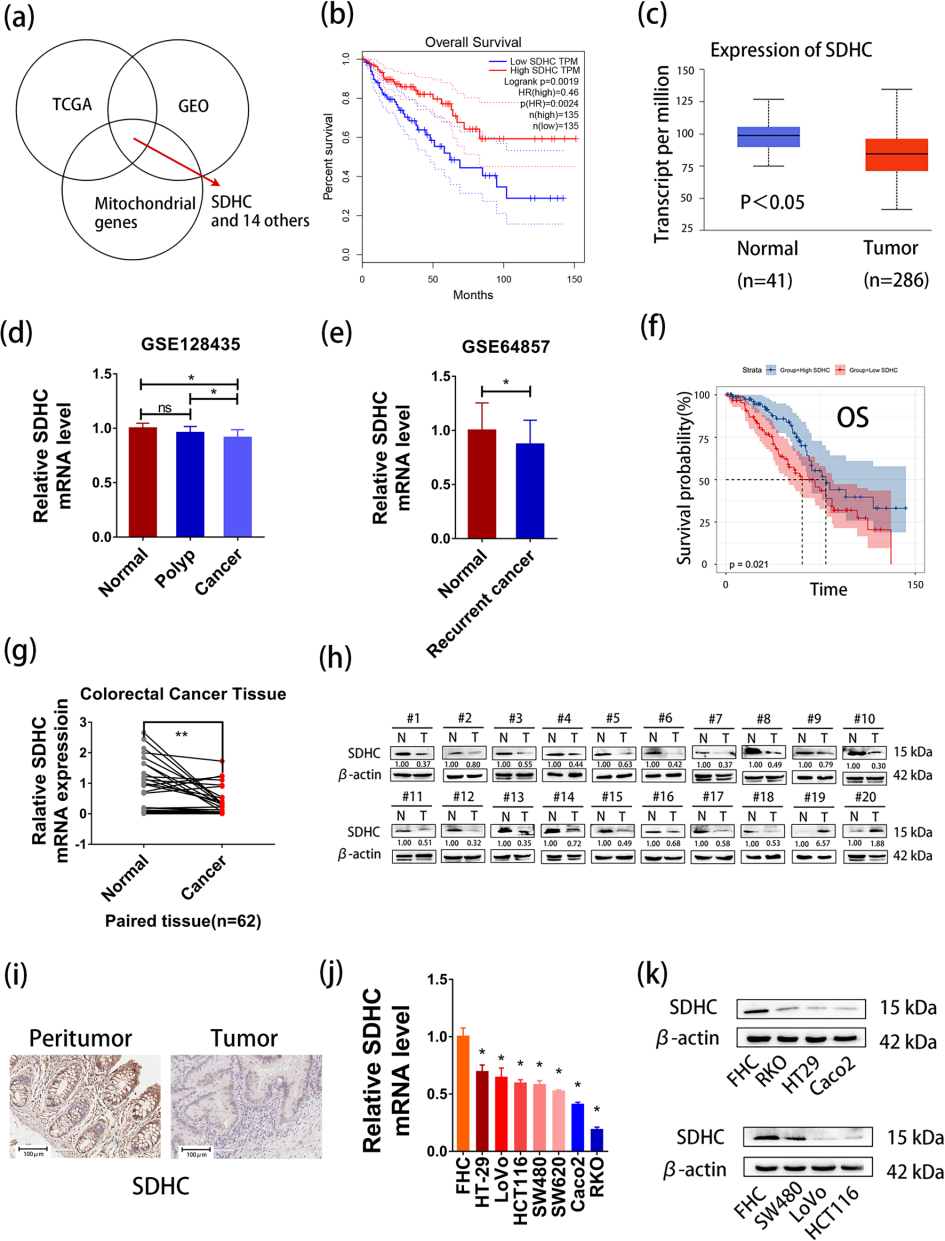
Expression and prognostic value of genes in colorectal cancer. **a** Intersection of differential genes in GEO, TCGA and mitochondrial genes. The dataset in GEO includes GSE81582, GSE77953, GSE77199 and GSE41258. **b** Low expression of SDHC was found to be associated with poor Overall Survival in CRC by GEPIA. **c** Low expression of SDHC in CRC tissues compared to normal tissues by UALCAN. **d** Low expression of SDHC in CRC tissues compared to normal tissues or polyps in GSE128435. **e** Low expression of SDHC in recurrent CRC compared to normal tissues in GSE64857. **f** Low expression of SDHC was found to be associated with poor Overall Survival in CRC in GSE17538. **g** qRT-PCR analysis of SDHC expression in 62 CRC patient samples. **h** Western blot analysis of SDHC expression in CRC tissues compared with adjacent normal tissues. **i** Immunohistochemistry analysis of SDHC expression in CRC tissues compared with adjacent normal tissues. **j** qRT-PCR analysis of SDHC expression in CRC cells and normal colon cells. **k** Western blot analysis of SDHC expression in CRC cells and normal colon cells. Data are presented as the mean ± SD and analyzed by Student’s t-test. *P < 0.05, **P < 0.01, ***P < 0.001

First, we used GEO datasets to search for datasets associated with CRC metastasis. We found 4 datasets, namely GSE81582, GSE77953, GSE77199 and GSE41258. GSE81582, GSE77953 and GSE77199 are related to liver metastasis and GSE41258 is related to liver and lung metastasis. By intersecting the differentially expressed genes of these 4 datasets, differential genes of colorectal cancer in the TCGA database and mitochondrial genes, we obtained 15 genes (Fig. 1a). We analyzed the prognostic significance of these genes in CRC using the GEPIA. The results showed that low expression of SDHC was associated with worse prognosis in CRC (Fig. 1b). The results from UALCAN indicate that mRNA levels and protein levels of SDHC are downregulated in colorectal cancer (Fig. 1c, Additional file: Figure S1a). To further investigate the expression of SDHC in colorectal cancer, we analyzed GES128435 and GSE64857. GSE128435 is related to colorectal polys and cancer, while GSE64857 is related to cancer recurrence. We found that the expression of SDHC was low in CRC (Fig. 1d–e). Moreover, the relationship between low expression of SDHC and worse prognostic in CRC was confirmed by GSE17538 (Fig. 1f, Additional file: Figure S1b).

The SDHC expression was measured in 62 patients with CRC using qRT-PCR. The results demonstrated a significantly lower expression of SDHC in CRC tissues compared to paired peri-tumor tissues (Fig. 1g). To validate this finding, Western blot and immunohistochemistry were employed, confirming the low expression of SDHC in CRC (Fig. 1h, i). Moreover, when compared to normal intestinal epithelial cells, various colorectal cancer cell lines exhibited low expression of SDHC at both mRNA and protein levels (Fig. 1j, k).

### SDHC regulates CRC cell migration and invasion

**Fig. 2:**
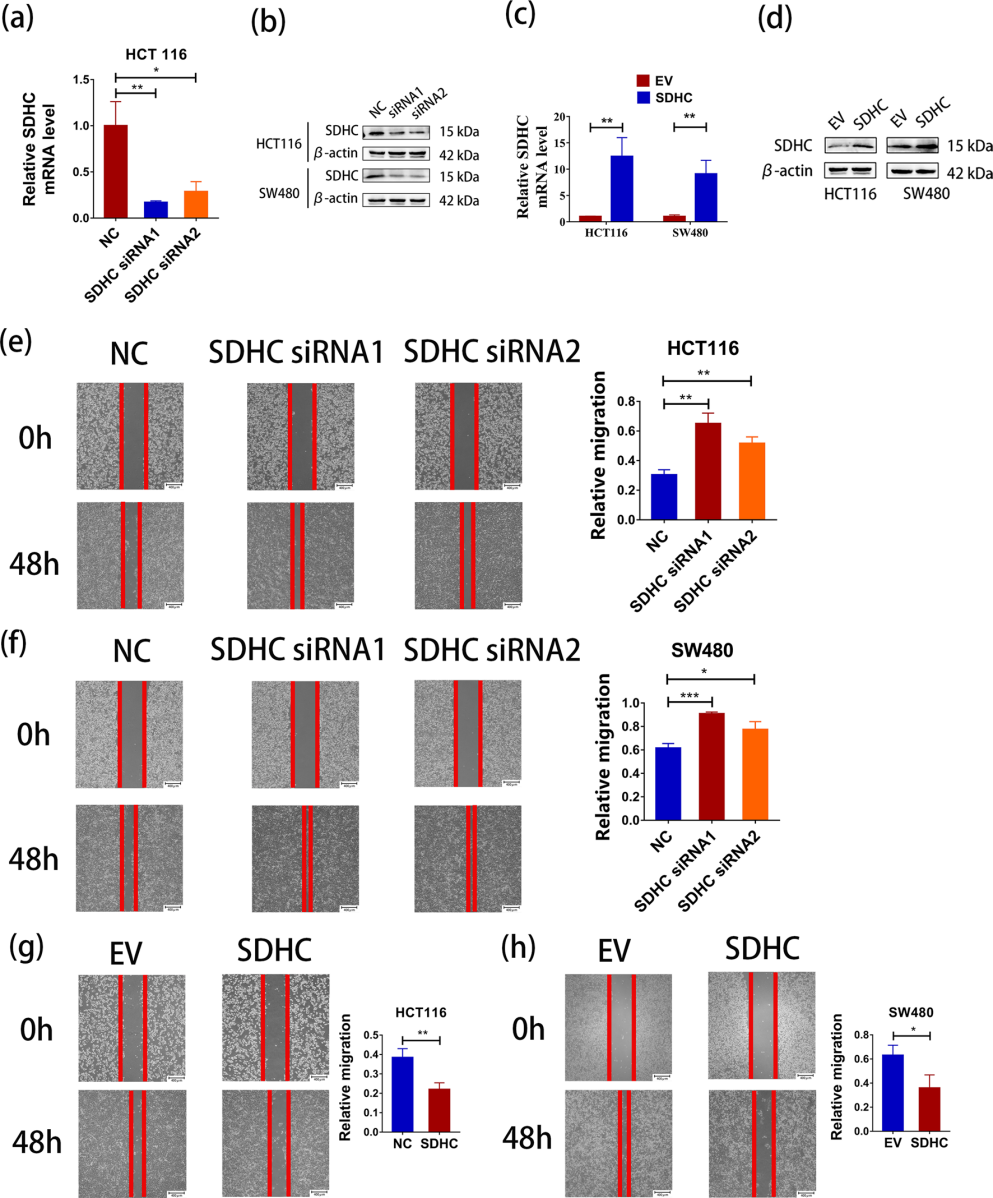
SDHC contributed to CRC metastasis and in vitro. **a** SDHC levels in HCT116 cells were analyzed by qPCR after transfection with siRNA negative control (NC), SDHC siRNA1 and SDHC siRNA2. **b** SDHC levels in HCT116 and SW480 cells were analyzed by western blot after transfection with NC, SDHC siRNA1 and SDHC siRNA2. **c** The overexpression of SDHC levels in HCT116 cells were analyzed by qPCR. **d** The overexpression of SDHC levels in HCT116 and SW480 cells were analyzed by western blot. **e** Wound healing assays showed that SDHC knockdown could promote the healing of scratches in HCT116 cells. **f** Overexpression of SDHC repressed the healing of scratches in HCT116 cells. **g** Wound healing assays showed that SDHC knockdown could promote the healing of scratches in SW480 cells. **h** Overexpression of SDHC repressed the healing of scratches in SW480 cells. Data are presented as the mean ± SD and analyzed by Student’s t-test. *P < 0.05, **P < 0.01, ***P < 0.001

**Fig. 3:**
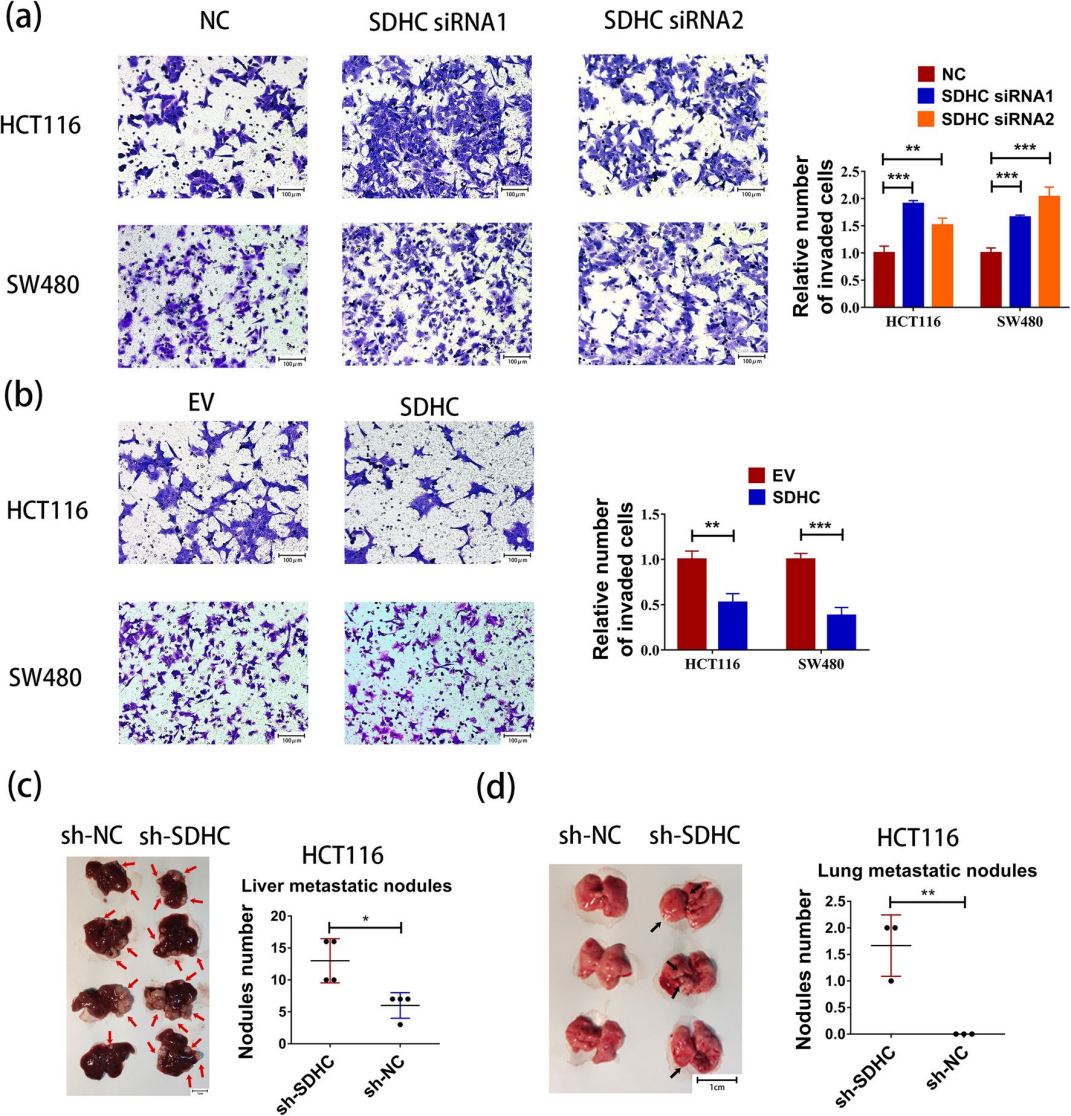
SDHC contributed to CRC metastasis in vitro. **a** Transwell assays revealed that SDHC knockdown promoted HCT116 and SW480 cells invasion. **b** Transwell assays revealed that overexpression of SDHC inhibited HCT116 and SW480 cells invasion. **c** Livers were excised from tumor-bearing mice and metastatic nodules were determined. Left panel shows representative liver tissues with metastatic nodules, the red arrows indicated the tumor nodules. Right panel, analysis of liver nodule numbers. Each dot denotes an animal. **d** Lung tumors were shown. The black arrows indicated the tumor nodules. And the number of metastasis in the lung was determined. Data are presented as the mean ± SD and analyzed by Student’s t-test. *P < 0.05, **P < 0.01, ***P < 0.001

The knockdown cell model of SDHC was established by transfecting HCT116 and SW480 cells with two siRNAs (SDHC siRNA1 and SDHC siRNA2). We confirmed the knockdown efficiency through qRT-PCR and western blot analysis (Fig. 2a, b, Additional file: Figure S1c). To establish the upregulation cell model of SDHC, we enhanced SDHC expression and assessed mRNA and protein levels to ensure high expression of SDHC (Fig. 2c, d). Wound healing assays revealed that SDHC knockdown significantly increased the migratory ability of HCT116 and SW480 cells, while overexpressing of SDHC in HCT116 and SW480 cells led to a significantly decrease in migratory capability compared to control cells (Fig. 2e–h). Additionally, transwell assays demonstrated enhanced invasion capacity in SDHC knockdown HCT116 and SW480 cells compared to control cells, whereas SDHC overexpressing HCT116 and SW480 cells results in reduced invasion capacity (Fig. 3a, b).

To further explore the potential role of SDHC in CRC in vivo, we injected HCT116 cells with stable SDHC knockdown (HCT116-sh-SDHC) into nude mice via splenic injection and counted metastatic foci in the mouse livers. We observed a significant increase in the number and size of metastatic nodules in the liver of the SDHC knockdown group compared to controls (Fig. 3c). Furthermore, to evaluate the biological function of SDHC in CRC lung metastasis, we intravenously injected HCT116-sh-SDHC cells into nude mice compared to a negative control group. Similarly, knockdown of SDHC enhanced CRC lung metastasis (Fig. 3d).

### Bioinformatic analysis of high-throughput RNA sequencing

**Fig. 4:**
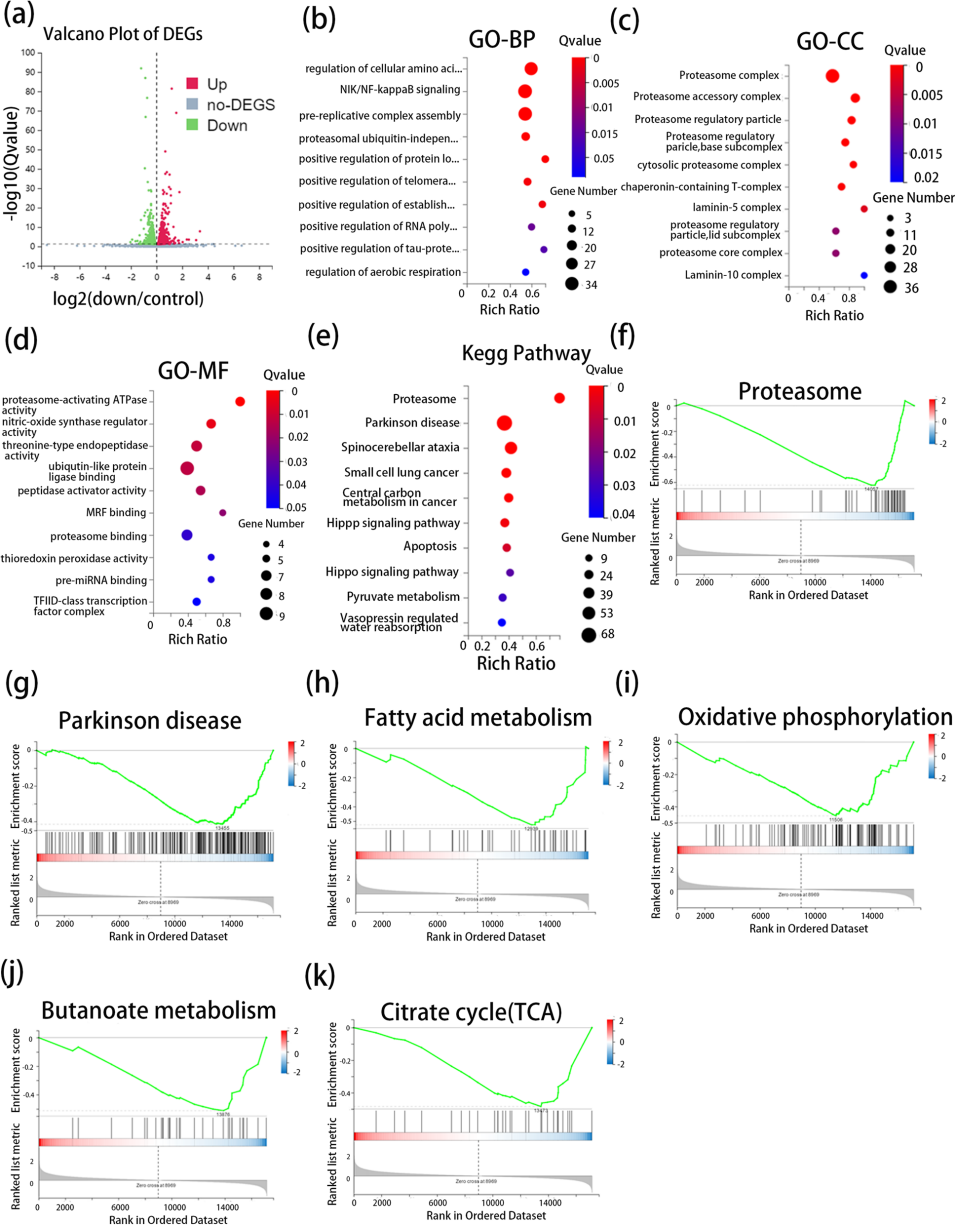
Bioinformatics analysis. **a** The volcano plot represents the DEGs. **b** GO annotations of the DEGs. The Bulb map presents enrichment scores of the top 10 significantly enriched GO terms in biological processes. **c** GO annotations of the DEGs. The Bulb map presents enrichment scores of the top 10 significantly enriched GO terms in cellular components. **d** GO annotations of the DEGs. The Bulb map presents the enrichment scores of the top 10 significantly enriched GO terms in molecular functions. **e** KEGG pathway enrichment analysis of DEGs. **f** Proteasome pathway enriched by GSEA. **g** Parkinson disease pathway enriched by GSEA. **h** Fatty acid metabolism pathway enriched by GSEA. **i** Oxidative phosphorylation pathway enriched by GSEA. **j** butanoate metabolism pathway enriched by GSEA. **k** Citrate cycle (TCA cycle) pathway enriched by GSEA. Data are presented as the mean ± SD and analyzed by Student’s t-test. *P < 0.05, **P < 0.01, ***P < 0.001

We used high-throughput RNA sequencing to identify differentially expressed genes (DEGs) between CRC cell lines knocked down for SDHC using sh-SDHC and CRC cell lines targeted with a scrambled shRNA. We screened DEGs using a threshold value with P < 0.05. The volcano plot presents the difference in mRNA expression between the two groups (Fig. 4a). We found 1779 DEGs of which 893 were positively correlated with SDHC and 886 were negatively correlated with SDHC. Next, GO and KEGG pathway analyses of DEGs were conducted to identify the pathways associated with these DEGs, GO analysis covers three domains: biological processes (BP), cellular components (CC), and molecular functions (MF). The top five terms of BP were positive regulation of protein localization to Cajal body, positive regulation of tau-protein kinase activity, positive regulation of establishment of protein localization to telomere, positive regulation of RNA polymerase II transcriptional preinitiation complex assembly, and regulation of cellular amino acid metabolic process (Fig. 4b). The top five terms of CC were laminin-5 complex, laminin-10 complex, proteasome accessory complex, cytosolic proteasome complex, and proteasome regulatory particle (Fig. 4c). The top five terms of MF were proteasome-activating ATPase activity, MRF binding, nitric-oxide synthase regulator activity, thioredoxin peroxidase activity, and pre-miRNA binding (Fig. 4d). The top five terms of KEGG pathway were Proteasome, Spinocerebellar ataxia, Hippo signaling pathway, Central carbon metabolism in cancer and Apoptosis (Fig. 4e). The results of GO and KEGG above can also be found in (Additional file: Table S1–S4). To further investigate the potential function of SDHC in CRC, we performed Gene-set enrichment analysis (GSEA) on RNA-seq data. The results were included Proteasome, Parkinson’s disease, Fatty acid metabolism, Oxidative phosphorylation, Butanoate metabolism and Citrate cycle (TCA cycle) (Fig. 4f–k, Additional file: Table S5).

### Lipid accumulation was mediated by SDHC silencing

**Fig. 5:**
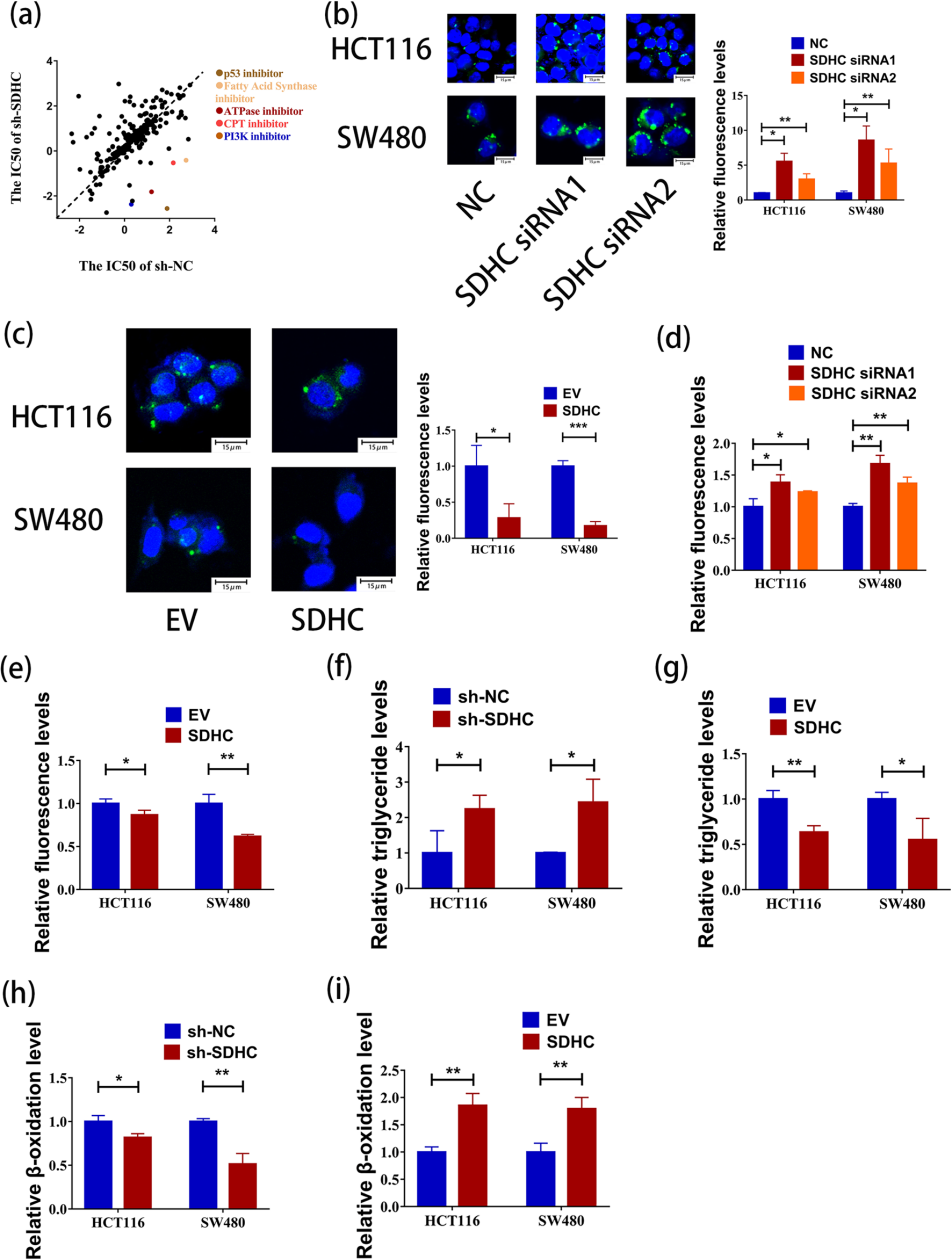
Lipid accumulation was mediated by SDHC silencing. **a** A log10-IC50 plot of the screening results. A log10 scale of IC50 values of the drugs against HCT116-sh-SDHC and control cells was plotted. Drugs with selectivity index (SI) > 2 were selected and marked as synthetic lethality candidates. **b** The content of neutral lipids in HCT116 (NC/siRNA1/siRNA2) and SW480 (NC/siRNA1/siRNA2) cells were measured by Confocal microscope. **c** The content of neutral lipids in HCT116 (EV/SDHC) and SW480 (EV/SDHC) cells were measured by Confocal microscope. **d** The content of neutral lipids in HCT116 (NC/siRNA1/siRNA2) and SW480 (NC/siRNA1/siRNA2) cells were measured by flow cytometry. **e** The content of neutral lipids in HCT116 (EV/SDHC) and SW480 (EV/SDHC) cells were measured by flow cytometry. **f** The silencing of SDHC enhanced the triglyceride levels in HCT116 and SW480 cells. **g** SDHC overexpression decreased the triglyceride levels in HCT116 and SW480 cells. **h** The silencing of SDHC decreased the FAO rate in HCT116 and SW480 cells. **i** SDHC overexpression increased the FAO rate in HCT116 and SW480 cells. Data are presented as the mean ± SD and analyzed by Student’s t-test. *P < 0.05, **P < 0.01, ***P < 0.001

**Fig. 6:**
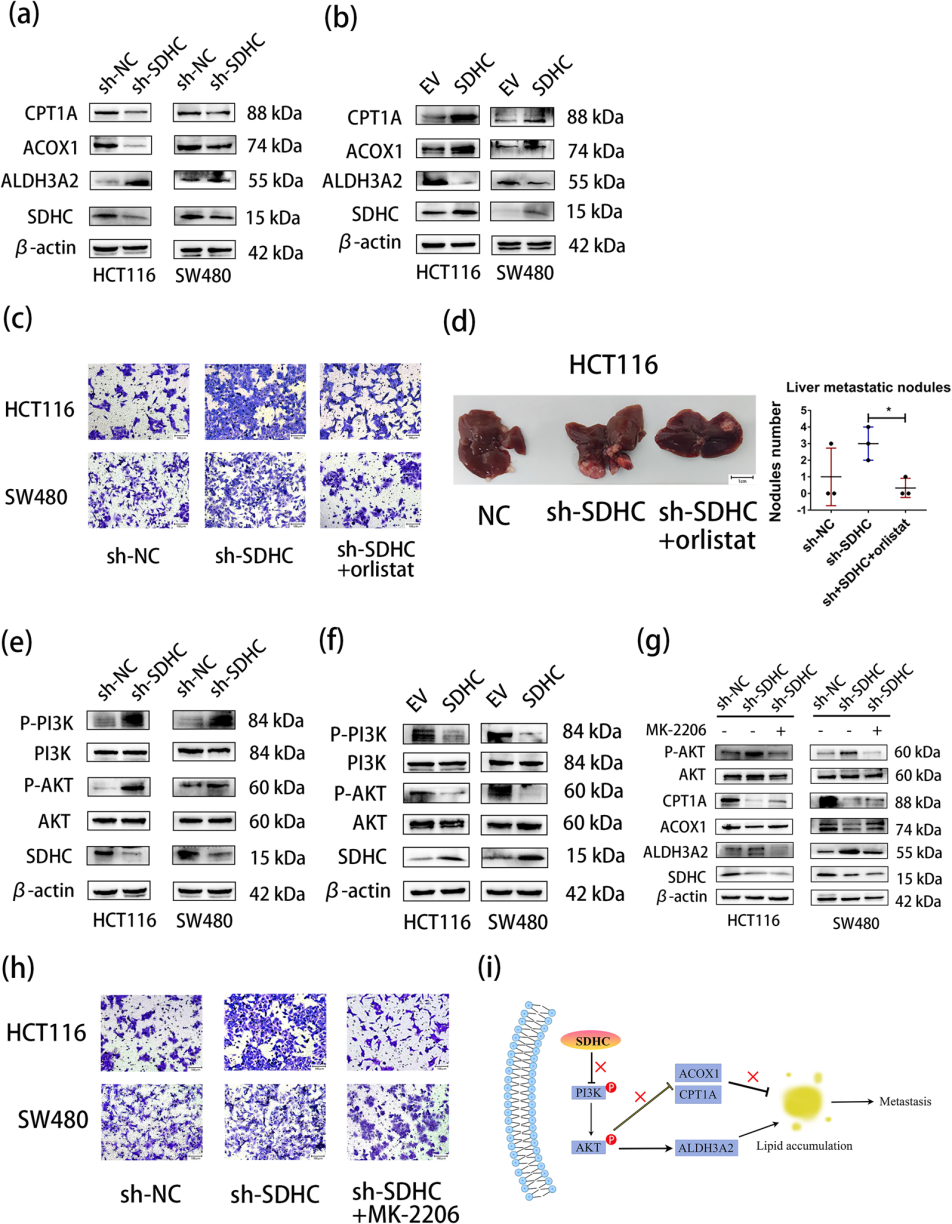
SDHC reprogramed fatty acid metabolism by regulating the PI3K/AKT pathways. **a** Western blotting was used to demonstrate the effects of silencing SDHC on the expression of critical enzymes involved in lipid metabolism, namely ACOX1, CPT1A, and ALDH3A2. **b** Western blotting was used to demonstrate the effects of overexpression SDHC on the expression of critical enzymes involved in lipid metabolism. **c** Migration assay analysis of orlistat in HCT116 and SW480 cells with SDHC decreased. **d** The effect of orlistat on liver metastasis formation in nude mice bearing HCT116 cells with SDHC decreased. **e** Western blotting reveals the levels of PI3K, p-PI3K, AKT and p-AKT in HCT116 and SW480 cells with SDHC decreased and control. **f** Western blotting demonstrates the levels of PI3K, p-PI3K, AKT and p-AKT in HCT116 and SW480 cells with SDHC overexpress and control. **g** Western blotting demonstrates the levels of AKT, p-AKT, ACOX1, CPT1A and ALDH3A2 in HCT116-sh-SDHC and SW480-sh-SDHC cells treated with MK-2206. **h** Migration assay analysis of MK-2206 in HCT116 and SW480 cells with SDHC decreased. **i** Schematic depiction of the regulation on fatty acid metabolism by SDHC in CRC cells. Data are presented as the mean ± SD and analyzed by Student’s t-test. *P < 0.05, **P < 0.01, ***P < 0.001

SDHC is an enzyme located in the mitochondria. According to our sequencing results, it is closely associated with metabolism. To investigate further, we conducted a screening using Anti-cancer Metabolism Compound Library consisting of 237 small-molecule inhibitors. The screening was performed in an 8-dose interplate titration format, using 384-well plates to determine the estimated IC50 values for each compound (Fig. 5a). Notably we discovered that inhibitors of fatty acid synthase were able to selectively kill SDHC knockdown cells. Additionally, when we examined the results of GSEA which indicated a positive correlation between fatty acid metabolism and SDHC (Fig. 4h), we predicted that SDHC is closely linked to lipid accumulation. To confirm the impact of SDHC on fatty acid metabolism, we conducted analyses of intracellular triglyceride content and FAO levels. Specifically, we used siRNA1 and siRNA2 to knock down SDHC in HCT116 and SW480 cells and labeled lipid droplets with the lipophilic dye BODIPY 493/503. Confocal microscope imaging demonstrated that cells with knocked-down SDHC exhibited larger and more lipid droplets (Fig. 5b). In contrast, SDHC overexpression decreased the intracellular lipid droplets (Fig. 5c). Flow cytometry further analysis revealed an increase in fluorescence intensity following SDHC knockdown, while SDHC overexpression had the opposite effects (Fig. 5d, e, Additional file: Figure S1d–g). Next, using a triglyceride detection kit, we observed an increase in cellular triglyceride content after SDHC knockdown (Fig. 5f). Conversely, when SDHC expression was elevated, the triglyceride content decreased (Fig. 5g). Additionally, we utilized a kit to measure the FAO rate in cells. The results indicated that knockdown of SDHC led to a decrease in FAO rate, signifying a slower fat metabolism (Fig. 5h). Conversely, when SDHC expression was elevated, the FAO rate increased (Fig. 5i). Furthermore, analysis of the GSEA data on key genes involved in the fatty acid metabolism pathway revealed that SDHC depletion resulted in reduced expression of ACOX1 and CPT1A, which are essential enzymes in fatty acid metabolism. Conversely, the expression of ALDH3A2, an enzyme that promotes fatty acid synthesis, increased. These findings were validated by confirming decreased expression of ACOX1 and CPT1A, as well as increased expression of ALDH3A2, after down-regulating SDHC (Fig. 6a, Additional file: Figure S1h–j). Conversely, when SDHC was overexpressed, we observed the opposite effects on these genes (Fig. 6b).

### SDHC reprogramed fatty acid metabolism by modulating the PI3K/AKT axes

In order to further confirm the impact of SDHC on tumor invasion and metastasis through lipid accumulation, we utilized orlistat, a fatty acid synthase inhibitor, to decrease the presence of lipid droplets in SDHC knockdown cells. The results from transwell experiments indicated that orlistat treatment reduced the effect of SDHC reduction on cell metastasis and invasion (Fig. 6c). We also verified this outcome using a mouse model, thereby confirming the crucial role of lipid accumulation in promoting liver metastasis of colorectal cancer cells (Fig. 6d).

We have identified 32 drugs with a selectivity index (SI) > 2. This included 6 drugs targeting the PI3K/AKT signaling pathway, 8 drugs targeting Metabolism, 6 drugs targeting Membrane transporter/Ion channel, 3 drugs targeting Apoptosis, 3 drugs targeting Angiogenesis and 2 drugs targeting Autophagy (Additional file: Table S6). We hypothesized that SDHC influences intracellular lipid accumulation through PI3K/AKT signaling pathway. Western blot analysis revealed an increase in the expression of phosphorylated PI3K and phosphorylated AKT following SDHC knockdown, while the total protein levels of PI3K and AKT did not exhibit significant changes (Fig. 6e). Consistent results were also observed in overexpressing SDHC (Fig. 6f). To inhibit AKT phosphorylation, we employed MK-2206, and observed the restoration of the down-regulation of ACOX1 and CPT1A, as well as the up-regulation of ALDH3A2 induced by SDHC down-regulation (Fig. 6g). Furthermore, MK-2206 effectively restrained the cell invasion caused by SDHC down-regulation (Fig. 6h).

## Discussion

No previous studies have comprehensively explored the roles and mechanisms of SDHC in CRC. In this study, we revealed that SDHC was downregulated in CRC and its low expression was linked to overall survival and disease-free survival, indicating that SDHC could be used as a prognosis predictor. Additionally, its low expression might predict a high risk of metastasis. To further investigate the involvement of SDHC in CRC, we conducted GSEA analysis and drug screening which suggested that SDHC might play a role in fatty acid metabolism. Subsequent in vitro and in vivo experiments were performed, confirming that dysregulation of SDHC indeed contributed to the metastasis of CRC by regulating fatty acid metabolism. Our findings indicated that the tumor suppressor SDHC could repress fatty acid synthesis by decreasing the expression of ALDH3A2 and simultaneously promote FAO by upregulating the FAO-related enzymes ACOX1 and CPT1A through the PI3K/AKT signaling axes. A schematic model summarizing our discoveries is shown in Fig. 6i.

Research has shown that lipid accumulation promotes the metastasis of various tumors [24–27]. ALDH3A2 is an enzyme responsible for detoxifying fatty aldehydes and generating 16- and 18-carbon fatty acids, which is involved in fatty acid synthesis [28]. High ALDH3A2 expression predicts poor prognosis in ovarian cancer [29]. Accompanied by an impairment of mitochondrial function, downregulated of lipid oxidation enzymes ACOX1 and CPT1A increases lipid Accumulation [27, 30–33]. ACOX1 is highly downregulated in CRC and it could suppress CRC progression by regulating palmitic acid reprogramming [34]. PTPRO could suppress CRC development and metastasis by modulating the MAPK/PPARα/ACOX1 pathways and reprogramming lipid metabolism [27]. Activating the PPARα/CPT1A axis alleviates lipid accumulation and inhibits cell proliferation and migration of clear cell renal cell carcinoma [35]. The miR-532-5p could drives nodal metastasis of cervical cancer through CPT1A-mediated lipid droplet accumulation [24]. All in all, increasing ALDH3A2 while reducing ACOX1 and CPT1A can improve lipid accumulation. Based on the results of our experiment, we found that SDHC silencing could reduce the expression of ACOX1 and CPT1A while increasing the expression of ALDH3A2. This consequently enhances lipid accumulation in CRC. It has been established that the activation of the PI3K/AKT signaling pathway affects lipid accumulation [36, 37]. The PI3K-AKT pathways are responsible for GDF11-induced lipid accumulation in liver cancer cells [38]. By interfering with the PI3K/AKT signaling pathway, ALM has the capability to inhibit adipogenesis [39]. Considering the results of drug screening, it is likely that SDHC may play its biological role by regulating PI3K/AKT pathway. We confirmed that SDHC could affect the phosphorylation of PI3K and AKT. To further investigate the relationship between SDHC, lipid accumulation and PI3K/AKT signaling pathway, we used the AKT phosphorylation inhibitor MK-2206. MK-2206 is a highly selective allosteric inhibitor of AKT currently in phase II studies for patients with refractory renal cell carcinoma [40], relapsed or refractory lymphoma [41], and advanced thoracic malignancies [42]. As a promising drug in clinical antitumor therapy, MK-2206 has been shown to inhibit lipid accumulation [43]. Importantly, previous studies have indicated that SDHC deficiency could activate the AKT pathway [6]. Our current research demonstrates that MK-2206 has the ability to restore lipase activity that has been affected by SDHC. Furthermore, the treatment with MK-2206 eradicated the enforced metastasis induced by SDHC suppression.

Consequently, we unearthed the roles of SDHC in CRC using compelling evidence and found that suppressing SDHC could boost the levels of ALDH3A2 while reducing the expression of ACOX1 and CPT1A in CRC cells through the activation of the PI3K/AKT signaling pathways. Consequently, the accumulation of lipids facilitated by ALDH3A2, ACOX1, and CPT1A could further facilitate the metastasis in CRC.

Despite its intriguing findings, the present study still has some limitations: (1) we have not explored the reason behind the downregulation of SDHC in CRC; (2) Further research is needed to understand the mechanism by which SDHC deficiency improves PI3K/AKT phosphorylation; (3) The potential drug inducing synthetic lethality in SDHC-deficient colorectal cancer cells requires further investigation. Future research may be carried out by constructing SDHC knockout mice, on the one hand, through chemically induced tumorigenesis to clarify the more comprehensive mechanism of the regulation of colorectal cancer development and progression under the condition of SDHC deficiency, on the other hand, through drug screening to find therapeutic methods for SDHC deficient CRC.

## Conclusion

In summary, our investigation explored the roles of SDHC in CRC, uncovering its potential as both a tumor suppressor and a prognostic predictor. Our results indicated that lipid accumulation, resulting from the PI3K/AKT pathways, plays a vital role in CRC metastasis when SDHC is silenced.

### Supplementary Information

Additional file 1.

## Data Availability

Upon reasonable request, data and materials supporting these findings in the present study will be available from the corresponding author.
