# Supplementary material for: Deficiency of SDHC promotes metastasis by reprogramming fatty acid metabolism in colorectal cancer
Source: J Transl Med. 2024 Jun 6;22:544. doi: 10.1186/s12967-024-05361-x (PMC11157952; doi:10.1186/s12967-024-05361-x)
Supplement: Supplementary file 1 — Additional file 1. [file 12967_2024_5361_MOESM1_ESM.docx]

**Additional file**


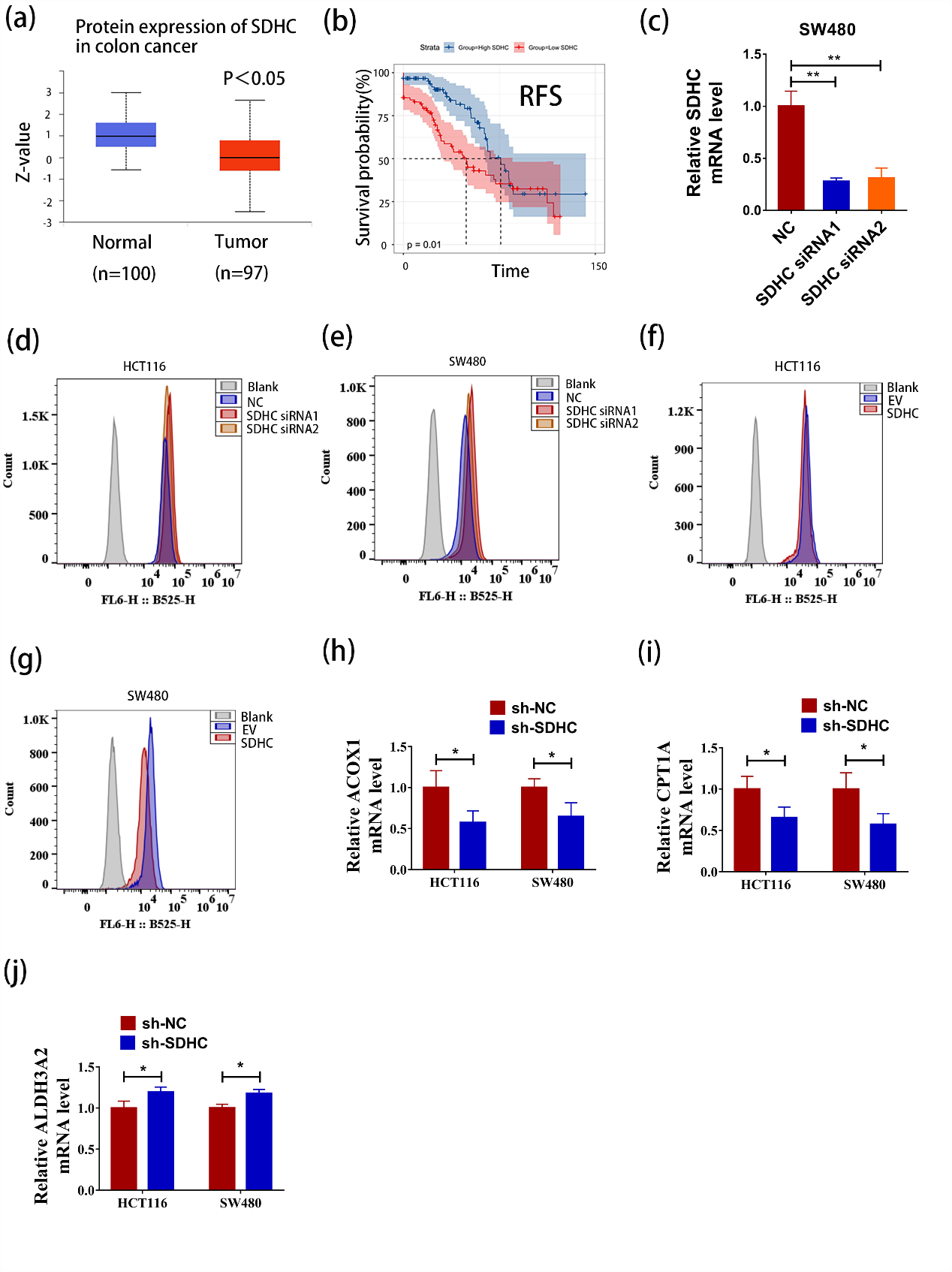


**Figure S1. (a)** The protein expression of SDHC was low in CRC compared to normal by UALCAN. **(b)** Low expression of SDHC was found to be associated with poor Recurrence free survival in CRC in GSE17538. **(c)** SDHC levels in SW480 cells were analyzed by qPCR after transfection with siRNA negative control (NC), SDHC siRNA1 and SDHC siRNA2. **(d-e)** Representative flow cytometry histograms showing the fluorescence for HCT116 (NC/ SDHC siRNA1/ SDHC siRNA2) and SW480 (NC/ SDHC siRNA1/ SDHC siRNA2) cells modified with BODIPY 493/503. Shown in gray is the fluorescence for unmodified control cells. **(f-g)** Representative flow cytometry histograms showing the fluorescence for HCT116 (EV/ SDHC) and SW480 (EV/ SDHC) cells modified with BODIPY 493/503. Shown in gray is the fluorescence for unmodified control cells. **(h-j)** qPCR was used to demonstrate the effects of silencing SDHC on the expression of critical enzymes involved in lipid metabolism, namely ACOX1, CPT1A, and ALDH3A2. Data are presented as the mean ± SD and analyzed by Student’s t-test. *P < 0.05, **P < 0.01, ***P < 0.001.

**Table S1.** Biological Processes

| Term ID | Term Description | Rich Ratio | P value | Q value |
| --- | --- | --- | --- | --- |
| GO:1904871 | positive regulation of protein localization to Cajal body | 0.727273 | 1.08E-06 | 1.86E-04 |
| GO:1902949 | positive regulation of tau-protein kinase activity | 0.714286 | 1.62E-04 | 0.015317 |
| GO:1904851 | positive regulation of establishment of protein localization to telomere | 0.7 | 8.08E-06 | 0.001205 |
| GO:0045899 | positive regulation of RNA polymerase II transcriptional preinitiation complex assembly | 0.6 | 1.33E-04 | 0.013213 |
| GO:0006521 | regulation of cellular amino acid metabolic process | 0.596154 | 1.14E-18 | 3.48E-15 |
| GO:1904874 | positive regulation of telomerase RNA localization to Cajal body | 0.5625 | 5.08E-06 | 7.99E-04 |
| GO:0010499 | proteasomal ubiquitin-independent protein catabolic process | 0.545455 | 1.99E-07 | 4.03E-05 |
| GO:1903715 | regulation of aerobic respiration | 0.545455 | 2.68E-04 | 0.022668 |

**Table S2.** Cellular Components

| Term ID | Term Description | Rich Ratio | P value | Q value |
| --- | --- | --- | --- | --- |
| GO:0005610 | laminin-5 complex | 1 | 8.14E-05 | 0.002364 |
| GO:0043259 | laminin-10 complex | 1 | 8.58E-04 | 0.018017 |
| GO:0022624 | proteasome accessory complex | 0.882353 | 5.01E-14 | 7.07E-12 |
| GO:0031597 | cytosolic proteasome complex | 0.857143 | 4.71E-06 | 2.33E-04 |
| GO:0005838 | proteasome regulatory particle | 0.833333 | 3.25E-09 | 3.21E-07 |
| GO:0008540 | proteasome regulatory particle, base subcomplex | 0.75 | 1.05E-07 | 7.96E-06 |
| GO:0005832 | chaperonin-containing T-complex | 0.7 | 6.43E-06 | 2.88E-04 |
| GO:0008541 | proteasome regulatory particle, lid subcomplex | 0.625 | 3.38E-04 | 0.007763 |

**Table S3.** Molecular Functions

| Term ID | Term Description | Rich Ratio | P value | Q value |
| --- | --- | --- | --- | --- |
| GO:0036402 | proteasome-activating ATPase activity | 1 | 8.02E-07 | 1.65E-04 |
| GO:0043426 | MRF binding | 0.8 | 3.99E-04 | 0.01993 |
| GO:0030235 | nitric-oxide synthase regulator activity | 0.666667 | 5.20E-05 | 0.004288 |
| GO:0008379 | thioredoxin peroxidase activity | 0.666667 | 0.001105 | 0.040479 |
| GO:0070883 | pre-miRNA binding | 0.666667 | 0.001105 | 0.040479 |
| GO:0016504 | peptidase activator activity | 0.545455 | 2.41E-04 | 0.014426 |
| GO:0004298 | threonine-type endopeptidase activity | 0.5 | 1.43E-04 | 0.010819 |
| GO:0001094 | TFIID-class transcription factor complex binding | 0.5 | 0.001385 | 0.045639 |

**Table S4.** KEGG Pathway

| Term ID | Term Description | Rich Ratio | P value | Q value |
| --- | --- | --- | --- | --- |
| 3050 | Proteasome | 0.673913 | 5.44E-20 | 1.84E-17 |
| 5017 | Spinocerebellar ataxia | 0.316901 | 6.54E-12 | 5.53E-10 |
| 4392 | Hippo signaling pathway - multiple species | 0.310345 | 0.002464 | 0.023003 |
| 5230 | Central carbon metabolism in cancer | 0.3 | 7.69E-06 | 2.00E-04 |
| 4214 | Apoptosis | 0.285714 | 4.40E-04 | 0.005955 |
| 5222 | Small cell lung cancer | 0.282609 | 2.29E-06 | 7.73E-05 |
| 4391 | Hippo signaling pathway | 0.271429 | 9.55E-05 | 0.001793 |
| 5012 | Parkinson disease | 0.268775 | 2.04E-13 | 3.46E-11 |

**Table S5.** GSEA

| MSigDB C2(curated) KEGG Term Desc | NES | NOM p-val | FDR q-val |
| --- | --- | --- | --- |
| Proteasome | 2.250839 | 0 | 0 |
| Parkinson's disease | 2.119942 | 0 | 0 |
| Oxidative phosphorylation | 1.937079 | 0 | 0.009484 |
| Fatty acid metabolism | 1.885555 | 0 | 0.012493 |
| Butanoate metabolism | 1.773249 | 0.005602 | 0.035317 |
| Citrate cycle (TCA cycle) | 1.653092 | 0.017094 | 0.091673 |

**Table S6.** Drugs screening

| **Drugs** | **Target** | **selectivity index (SI)** |
| --- | --- | --- |
| GSK1059615 | PI3K/AKT signaling | 470.478 |
| Tacrolimus | PI3K/AKT signaling | 56.32805 |
| Voxtalisib Analogue | PI3K/AKT signaling | 17.37569 |
| Adenosine 5'-monophosphate | PI3K/AKT signaling | 4.579134 |
| INK 128 | PI3K/AKT signaling | 2.921348 |
| Temsirolimus | PI3K/AKT signaling | 2.500308 |
| P7C3 | Others | 87.08972 |
| Clotrimazole | Microbiology&Virology | 3.355631 |
| Pralatrexate | Metabolism | 374.7693 |
| Orlistat | Metabolism | 1390.484 |
| (R)-Etomoxir sodium salt | Metabolism | 497.6239 |
| Mevastatin | Metabolism | 7.615274 |
| Prednisolone-21-acetate | Metabolism | 3.933708 |
| Atorvastatin Calcium | Metabolism | 2.796075 |
| Sulfameter | Metabolism | 2.390029 |
| Tioxolone | Metabolism | 2.115538 |
| Cinobufagin | Membrane transporter/Ion channel | 1042.735 |
| Ranolazine dihydrochloride | Membrane transporter/Ion channel | 50.32815 |
| Revaprazan hydrochloride | Membrane transporter/Ion channel | 8.625819 |
| CFTRinh172 | Membrane transporter/Ion channel | 6.00951 |
| Esomeprazole Sodium | Membrane transporter/Ion channel | 2.802787 |
| Sudoterb free base | Membrane transporter/Ion channel | 2.349109 |
| TBHQ | Immunology/Inflammation | 3.350621 |
| Curcumin | Chromatin/Epigenetic; Others | 15.77938 |
| Gimeracil | Autophagy | 205.0812 |
| Sulfanilamide | Autophagy | 8.325187 |
| Pifithrin-μ | Apoptosis | 27753.78 |
| PK11007 | Apoptosis | 4.109632 |
| COTI2 | Apoptosis | 2.79421 |
| LW6 | Angiogenesis | 2.66004 |
| IDF-11774 | Angiogenesis | 74.21094 |
| Lificiguat | Angiogenesis | 3.042985 |
